# Prosthetic Dentistry

**Published:** 1898-09

**Authors:** Alvin J. Hovey

**Affiliations:** Mt. Vernon, Ind.


### ARTICLE VII.

### PROSTHETIC DENTISTRY.

DR. ALVIN J. HOVEY, MT. VERNON, IND.

Read before the Evansville Dental Society, June 20th, 1898.

We find in most all professions and trades certain divisions and limitations in which specialties become prime factors and instigators.

The medical profession is one which contains numerous branches of grave importance.

Dentistry is one of the principal specialties of medicine, and when we go still farther we find that dentistry itself is divided into two branches of very prominent importance—mechanical and operative, or "prosthetic and surgical."

Prosthetic dentistry to a great extent cannot be acquired; operative dentistry to a great extent cannot be acquired. A person can be taught and become a satisfactory operator without any natural mechanical instincts; at the same time, while mechanism will be of great assistance to an operator, it is not an essential necessity; while on the other hand a person must be gifted with a certain amount of mechanical ingenuity to even meet with partial success. Most any dentist can extract a tooth, but there are not so many who can properly place a substitute. Most any dentist can amputate a tooth, but again there are not so many who can attach another.

As a rule, at the present time numbers of teeth can be extracted without any great difficulty, but when it comes to replacing those teeth with a plate, it requires thought, time and labor, and draws on one's ingenuity, skill and exactness.

The mechanical department of dentistry forms the great bulk of work of this profession. All dentists are not mechanics, but most persons with mechanical inclinations and requirements make good dentists. A person may be good at the chair, with the chisel, forceps and plugger, but he may not be able to successfully construct a plate, and he can only be taught to a certain extent.

Now, on the other hand, he may be prosthetically perfect, his mechanical skill may be unexcelled, and still he may be unable to properly insert a filling, but give him time and proper training, and he will soon learn.

In making full and partial dentures there are always new difficulties arising and requirements to be met, which necessitate no little attention. We never find two mouths alike; there is certain to be a difference. There is as great a variety of mouths as there are shells on the seashore.

While I have had very sattsfactory results from my plate work, although my experience in this line has been somewhat limited, I believe that plate work is the most unsatisfactory work of the dentist. You are not only compelled to satisfy the desires of you patients, but as a rule, also their aunts, uncles, cousins, brothers, sisters and nephews. They will say the teeth are too long, too short, too dark, etc., etc. Some one will say: "Why, doctor, the teeth overlap the lower ones," and some one with a ratbite will say: "The teeth don't strike like mine." There are a hundred and one difficulties to overcome. While technicalities may not be of such great importance in prosthetic work as in operative, they should be fully observed to be able to meet with success. While skill and ability are most important factors in prosthetic work, time is also most important. There are some portions of mechanical work which cannot be rushed. Plaster must be given time to harden, set and dry. Vulcanizing and investing each require a certain length of time. A great many of us neglect the prosthetic side of our profession for more reasons than one. One is that it is dirty and makes our hands unfit for the chair, and another is that it obstructs and interferes with the operative work. But when our business increases to such an extent that it interferes with our operations at the chair, we should lose no time in employing an assistant. An assistant will create more expense, but in the long run it will be found that it will pay. We make a mistake in overworking ourselves. Overwork breaks our health, and poor health is an obstacle of success.

Dentistry is not only a specialty of medicine, but to-day we find specialties in dentistry. There are dentists of to-day who make a specialty of fillings; there are some who do nothing but extraction, and there are some who put their time on mechanical work.

Though my experience in dentistry is limited, I would say to those who are starting out in our profession to strictly observe and adhere to the prosthetic branch. Even if you are born with natural inclinations for this work, don't think you know it all. It is hard at the easiest, and needs our strict attention.

In the prosthetic work of dentistry we deal with numerous materials. Plaster is one of the greatest necessities of the mechanical man. Without it he could not work. He needs it at almost every move and turn, and it would be a difficult task to find a substitute. It has several properties which should not be overlooked, such as expansion, absorption and the generation of heat. Plaster in setting is like time—it waits for no one. It should be kept in a dry place, and, if stirred and mixed to a medium consistency, will give best results. I use plaster in taking all impressions, and do not see how it could be beaten. I will not speak of other materials, as there are so many it would require all night.

In the restoration of the mouth by artificial dentures the absence of the individual functions of each tooth or tissue must not be neglected. Each natural tooth is placed there with its respective functions to perform, and each tooth not only performs its given functions, but is there to assist and protect its constituents in the natural formation of the mouth. Therefore, in making substitutes for the natural organs, it is our duty to construct such substitutes with the view of perfecting the lost tissues or improving the condition of the surroundings. The strength of a denture depends greatly upon the individual and the surrounding conditions. Long jaws, short jaws, crooked jaws and broken jaws will govern the size and strength of the denture to a great extent.

A man to become scientifically prosthetic in the building of dentures should be thoroughly acquainted with the muscles of action and expression; he should have a clear conception of the abnormalities which may exist, and be able to combat and restore to them their proper condition. Individual functions help and build uniformal function. Without the respective individual help of each tooth or structure uniformity cannot exist.

In this, the prosthetic branch of dentistry, we cannot sleep, and our judgment is always at stake.—*Indiana Dental Journal.*

